# Whole Exome Sequence Analysis Provides Novel Insights into the Genetic Framework of Childhood-Onset Pulmonary Arterial Hypertension

**DOI:** 10.3390/genes11111328

**Published:** 2020-11-11

**Authors:** Simone M. Gelinas, Clare E. Benson, Mohammed A. Khan, Rolf M. F. Berger, Richard C. Trembath, Rajiv D. Machado, Laura Southgate

**Affiliations:** 1 Genetics Research Centre, Molecular and Clinical Sciences Research Institute, St George’s University of London, London SW17 0RE, UK; simone.gelinas@gstt.nhs.uk (S.M.G.); p1805584@sgul.ac.uk (C.E.B.); m1500901@sgul.ac.uk (M.A.K.); 2 Center for Congenital Heart Diseases, Department of Pediatric Cardiology, Beatrix Children’s Hospital, University Medical Center Groningen, 9700 RB Groningen, The Netherlands; r.m.f.berger@umcg.nl; 3 Department of Medical & Molecular Genetics, Faculty of Life Sciences & Medicine, King’s College London, London SE1 9RT, UK; richard.trembath@kcl.ac.uk; 4 Institute of Medical and Biomedical Education, St George’s University of London, London SW17 0RE, UK

**Keywords:** exome sequencing, molecular genetics, lung disease, paediatrics, pulmonary arterial hypertension

## Abstract

Pulmonary arterial hypertension (PAH) describes a rare, progressive vascular disease caused by the obstruction of pulmonary arterioles, typically resulting in right heart failure. Whilst PAH most often manifests in adulthood, paediatric disease is considered to be a distinct entity with increased morbidity and often an unexplained resistance to current therapies. Recent genetic studies have substantially increased our understanding of PAH pathogenesis, providing opportunities for molecular diagnosis and presymptomatic genetic testing in families. However, the genetic architecture of childhood-onset PAH remains relatively poorly characterised. We sought to investigate a previously unsolved paediatric cohort (*n* = 18) using whole exome sequencing to improve the molecular diagnosis of childhood-onset PAH. Through a targeted investigation of 26 candidate genes, we applied a rigorous variant filtering methodology to enrich for rare, likely pathogenic variants. This analysis led to the detection of novel PAH risk alleles in five genes, including the first identification of a heterozygous *ATP13A3* mutation in childhood-onset disease. In addition, we provide the first independent validation of *BMP10* and *PDGFD* as genetic risk factors for PAH. These data provide a molecular diagnosis in 28% of paediatric cases, reflecting the increased genetic burden in childhood-onset disease and highlighting the importance of next-generation sequencing approaches to diagnostic surveillance.

## 1. Introduction

Pulmonary arterial hypertension (PAH) is an uncommon vascular disorder that remains incurable despite significant advances in current treatment regimens. PAH is characterised by obstruction and occlusion of the pulmonary arterioles leading to progressive pulmonary arterial pressure overload, right ventricular hypertrophy and failure of the right side of the heart [1]. Typical histopathological features of PAH include marked vascular remodelling of the pulmonary arterioles as a consequence of exuberant proliferation of pulmonary artery endothelial (PAEC) and smooth muscle (PASMC) cells. In families, PAH predominantly segregates as an autosomal dominant trait displaying features of complex disease, namely variable expressivity, reduced penetrance and a gender bias favouring females [2]. Despite major advances in delineating PAH aetiology, the associated morbidity and early mortality burden of this disease remains perniciously high, with an average life expectancy of 3–5 years from diagnosis [3].

Paediatric PAH represents a clinically distinct form of disease. Although less prevalent, at an estimated 4.8–8.1 cases/million [4,5], than the adult form (15–50 cases/million) [6], childhood-onset PAH (cPAH) is marked by greater morbidity, depressed responses to therapy and poor survival metrics [7,8]. Taken together, these features suggest a specific genetic background. Putatively causal genetic variants have been described in at least 26 PAH risk genes [9,10,11,12,13,14], of which deleterious variation in *BMPR2*, encoding a type II receptor of the transforming growth factor beta (TGF-beta) superfamily, was firmly established as the major risk factor in adult PAH [13]. In paediatric cases, rare deleterious mutations of *BMPR2* and other key components of the bone morphogenetic protein (BMP) signalling pathway have been determined to be rare causal factors. These include the receptor proteins activin A receptor-like type 1 (*ACVRL1*, encoding ALK1), *BMPR1B* and endoglin (*ENG*), as well as *GDF2* (encoding the ligand BMP9) and the cytoplasmic signalling mediator *SMAD9* [8,15,16,17,18,19,20].

Whole exome sequencing (WES) has been instrumental in the accelerated gene discovery in PAH, notably in genetic risk unrelated to the canonical BMP pathway. Moreover, these findings both expanded the mutation spectrum in cPAH and shed light on the epidemiology of this discrete disease entity. For example, mutations within the transcription factor genes T-box 4 (*TBX4*) and SRY-box 17 (*SOX17*) show enriched contribution to paediatric PAH compared to adult-onset, explaining up to 7.7% and 3.3% of paediatric cases, respectively [8,21,22]. Of note, *SOX17* mutations show a strong correlation to PAH associated with congenital heart disease in both adult and paediatric disease [22,23]. More recent findings include loss-of-function variants in the ATP-binding cassette subfamily C member 8 (*ABCC8*) gene and in the endothelial cell-specific ligand BMP10 [24,25], whilst genome-wide gene burden approaches in predominantly adult cohorts identified the candidate risk genes ATPase 13A3 (*ATP13A3*), fibulin 2 (*FBLN2*) and platelet-derived growth factor D (*PDGFD*), among others [10,14]. These novel gene discoveries demonstrated the benefit of a granular approach to the genomic interrogation of discrete case cohorts.

While these data further developed the molecular architecture of PAH, the mutational landscape remains relatively poorly understood in paediatric cohorts, particularly for more recently reported genes [10,12]. Here, we sought to investigate a previously unsolved paediatric cohort, first, to better define the mutational background of cPAH, and second, to augment the existing body of data underlying this disease. Taken together, this study highlights the hitherto undefined role of *ATP13A3* in the pathogenesis of paediatric PAH and, importantly, provides independent validation of putative gene-disease associations in *BMP10* and *PDGFD*.

## 2. Materials and Methods

### 2.1. Patient Cohort and Clinical Assessment

Eighteen unrelated patients and their relatives were recruited from specialist PAH centres in the UK and the Netherlands. Ethical approval for the study was received from local ethics review boards and written informed consent was obtained from all participants or their legal guardian. The study was conducted in accordance with the Declaration of Helsinki, and the protocol was approved by the St Thomas’ Hospital Research Ethics Committee (08/H0802/32).

Clinical diagnoses were established by PAH specialists in accordance with the classification of the World Symposium on Pulmonary Hypertension [26]. All patients previously underwent conventional genetic screening for *BMPR2*, *ACVRL1* and *ENG*, and were found to be mutation-negative [16,27]. Following pedigree analysis and clinical examination, patients were classified as having either idiopathic PAH (IPAH; *n* = 8), PAH associated with congenital heart disease (APAH-CHD; *n* = 7) or heritable PAH with one or more affected relatives (HPAH; *n* = 3).

### 2.2. Exome Sequencing

Genomic DNA was extracted from peripheral blood using standard methodologies. Whole exome sequencing for all probands was performed by Novogene Ltd. Briefly, exome libraries were captured using the SureSelect Human All Exon kit (Agilent Technologies, Santa Clara, CA, USA), and enriched fragments underwent paired-end sequencing on an Illumina platform. Quality control filtration of raw FASTQ files discarded read pairs containing adapter contamination and those with <80% of base calls achieving a Phred score of Q30. Sequence alignment to human reference genome assembly GRCh37 was undertaken using Burrows–Wheeler Aligner [28] and Picard MarkDuplicates (Genome Analysis Tool Kit (GATK), Broad Institute, Cambridge, MA, USA) was used to flag PCR duplicate reads for removal. GATK (v3.8) software was used for single nucleotide variant (SNV) and insertion and deletion (indel) detection [29]. Variant annotation was performed using ANNOtate VARiation (ANNOVAR) [30]. 

### 2.3. Candidate Gene Analysis

Annotated WES profiles for each patient were screened for rare, deleterious variants in 26 established and emerging PAH risk genes: *ABCC8*, *ACVRL1*, *AQP1*, *ATP13A3*, *BMP10*, *BMPR1B*, *BMPR2*, *CAV1*, *EIF2AK4*, *ENG*, *EVI5*, *FBLN2*, *GDF2*, *GGCX*, *KCNA5*, *KCNK3*, *KDR*, *KLF2*, *KLK1*, *NOTCH3*, *PDGFD*, *SMAD1*, *SMAD4*, *SMAD9*, *SOX17* and *TBX4*. Rare variants were defined as those with a minor allele frequency of ≤1 in 10,000 (MAF ≤ 0.0001), as reported in the Genome Aggregation Database control population (gnomAD v2.1.1; https://gnomad.broadinstitute.org). Splice region and exonic variants with likely functional consequences (nonsense, frameshift, nonframeshift indels and missense SNVs) were retained. Copy number variants were not considered in the analysis presented herein. The functional impact of missense variants was informed by in silico pathogenicity predictions. Specifically, variants with a Combined Annotation Dependent Depletion (CADD) score of ≥20 (https://cadd.gs.washington.edu) were considered potentially causal [31]. Additional evidence for pathogenicity was obtained from observing at least one deleterious score across Sorting Intolerant from Tolerant (SIFT; ≤0.05), PolyPhen-2 (≥0.909) and MutationTaster2 (annotation “disease causing”) algorithms [32,33,34]. The location of each variant was inspected for correlation with conserved protein domains, providing further confirmation of likely pathogenicity.

Candidate variants were visualised and inspected for artefacts using Integrative Genomics Viewer (IGV) to exclude false positives [35,36]. All identified variants were validated in an independent sample by Sanger sequencing (Eurofins Genomics, Ebersberg, Germany) and tested for familial co-segregation where DNA samples were available.

## 3. Results

### 3.1. WES Analysis of the cPAH Cohort

Overall, 92% of base calls achieved a mean quality score of Q30. A mean depth of coverage of 65x (range: 54x–78x) was achieved for all samples, with an average of 91% (range: 86–94%) of bases achieving >20x coverage. Manual inspection of all PAH risk genes using IGV confirmed sufficient coverage of ≥10x across all exonic regions and intron–exon boundaries.

A targeted variant analysis of previously reported risk genes in our panel of childhood-onset IPAH, APAH and HPAH patients revealed putatively pathogenic missense variants in *ABCC8*, *ATP13A3*, *BMP10*, *PDGFD* and *SMAD9* (Table 1). All variants were validated by Sanger sequencing. Based on our exclusion criteria, no other likely causal variants in the reported PAH risk genes were identified in these patients.

### 3.2. Novel Association of ATP13A3 with Paediatric APAH-CHD

**Figure 1:**
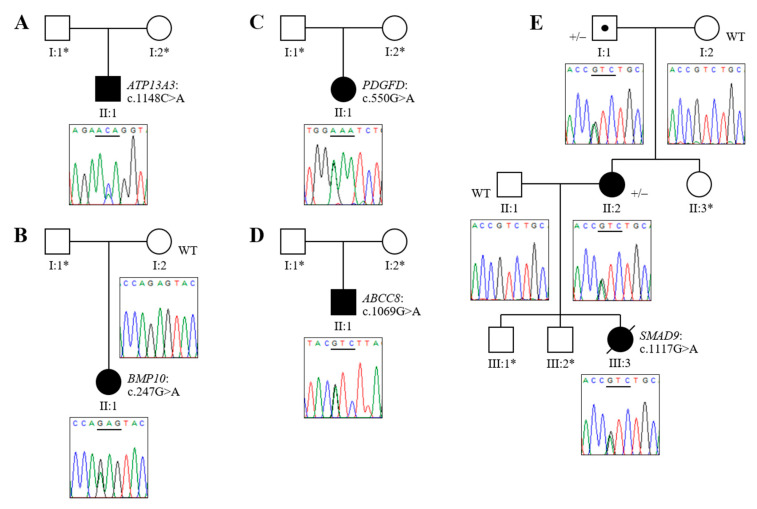
Sequence chromatograms and familial segregation of identified variants in childhood-onset pulmonary arterial hypertension (cPAH). (**A**) Patient 1 has a c.1148C>A missense mutation in *ATP13A3*; (**B**) Patient 2 has a heterozygous *BMP10* c.247G>A mutation; (**C**) Patient 3 is heterozygous for a c.550G>A mutation in *PDGFD*; (**D**) Patient 4 has a c.1069G>A missense variant in the *ABCC8* gene; (**E**) Patient 5 carries a heterozygous *SMAD9* c.1117G>A missense variant, which co-segregates with the disease (+/−) in his affected mother and maternal grandfather, who is an obligate carrier. Unaffected family members available for testing are all wild-type (WT). The horizontal line underlines the mutated codon. * DNA sample not available.

We identified a novel missense variant in *ATP13A3* (c.1148C>A, p.Thr383Lys) in a male child with APAH–CHD. To the best of our knowledge, this represents the first finding of a molecular defect in this risk gene in a paediatric case. The patient was diagnosed with PAH at nine years of age, associated with a secundum atrial septal defect (Figure 1A). All in silico predictions were consistent with a high probability of pathogenicity (Table 1). Parental samples were not available to conduct familial segregation analysis. 

While annotated as a missense variant, further analysis of the *ATP13A3* c.1148C>A variant revealed that the mutation is located 3 bp from the intron–exon boundary and one nucleotide upstream of the highly conserved donor splicing motif of exon 12 (CAG|gtagga). An independent interrogation of in silico data using the NNSplice tool in MutationTaster revealed a potential disruption of the donor splice site, likely resulting in the formation of an aberrant gene transcript or premature stop codon leading to nonsense-mediated decay of the messenger RNA. However, further work is required on the cDNA level to experimentally validate this predicted deleterious effect.

### 3.3. Novel Mutations of BMP10, PDGFD and ABCC8 in Childhood IPAH

In the IPAH cohort we detected a *BMP10* variant (c.247G>A, p.Glu83Lys) in a female Dutch patient diagnosed with severe disease at 36 months. The patient was stably treated on oral dual therapy (bosentan and sildenafil) but died at 18 years of age. This mutation, which is observed in 1/109,376 control individuals in gnomAD and was not previously associated with disease in a PAH clinical setting, provides the first independent confirmation of *BMP10* as a PAH risk gene [25]. All bioinformatic predictions were consistent with a high likelihood of pathogenicity (Table 1). Familial co-segregation analysis confirmed the unaffected mother was wild-type but, as no DNA was available from the father, it was not possible to determine whether this variant had arisen de novo in the proband (Figure 1B).

*PDGFD* is a newly identified risk gene in PAH [14]; this analysis offers the first independent validation of its causal association with PAH, as well as confirming a specific role in the development of paediatric disease through the identification of a rare missense variant (c.550G>A, p.Glu184Lys) in a female cPAH patient (Figure 1C). The variant, which is not present in population control databases, gave a CADD score of 28.2 and was predicted to be deleterious by all three missense prediction algorithms (Table 1).

A heterozygous missense variant in *ABCC8* (c.1069G>A, p.Val357Ile), novel in the context of PAH, was observed in a male patient diagnosed at 19 months of age (Figure 1D). While the CADD score and MutationTaster prediction were strongly indicative of a deleterious impact, the conclusions of pathogenicity based on other in silico markers were ambiguous, likely due to the interchangeability of valine and isoleucine residues at this position across related proteins in lower organisms (Table 1). Unavailability of parental DNA samples precluded familial segregation analysis of the *PDGFD* and *ABCC8* variants.

### 3.4. Analysis of HPAH Samples Identifies a Potential Molecular Defect of SMAD9

An examination of WES data from the three HPAH probands revealed a missense variant in *SMAD9* (c.1117G>A, p.Val373Ile) in a single family. Following a diagnosis of PAH, the proband died at five years of age. The affected amino acid residue displays high conservation across species, reflected in high deleterious scores across all in silico prediction algorithms employed (Table 1). Co-segregation was confirmed in two affected family members and an obligate carrier (Figure 1E). In addition, the candidacy of this variant as a causal defect in PAH was underpinned by the same finding in an HPAH patient reported in the ClinVar database (SCV000384082). However, as an observed allele frequency of >0.0001 in European controls exceeded our threshold for inclusion, this was resultantly considered to be a variant of unknown significance (VUS) in the absence of compelling functional data.

## 4. Discussion

This study applied a targeted 26 gene analysis to WES data in a European paediatric cohort, elucidating candidate molecular mechanisms in five patients to further illuminate the genetic architecture of early-onset PAH. It must be noted that all candidate variants presented in this analysis were categorised as variants of unknown significance in accordance with American College of Medical Genetics and Genomics (ACMG) guidelines [37], including those that met our criteria as being highly deleterious. Evidence of variant pathogenicity derived from population frequency, bioinformatic predictions and the biological relevance of these genes to PAH pathology is considered herein.

**Figure 2:**
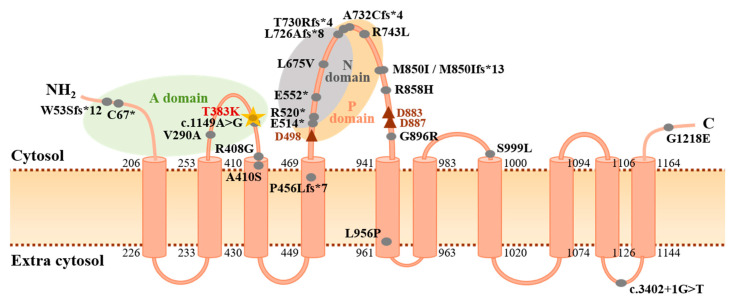
Protein structure of ATP13A3 highlighting mutations identified in PAH. Topological analysis of ATP13A3 according to UniProtKB protein component and site position data (ID: Q9H7F0) and published reports [38,40]. Likely pathogenic PAH mutations (CADD ≥ 15) reported in the literature are indicated by the filled grey circles [10,12,20,41]. The c.1148C>A (p.T383K) mutation identified in this study is highlighted by the gold star and is located adjacent to a previously reported splice-region variant [10]. Numbers indicate amino acid positions at each end of the 10 transmembrane domains. The red triangles denote essential asparagine residues (D498: active catalytic site; D883, D887: Mg^2+^ binding sites). A domain: actuator domain; C: carboxyl terminus; N domain: nucleotide binding domain; NH_2_: amino terminus; P domain: phosphorylation domain.

While *ATP13A3* was recently identified and functionally characterised as being causative in adult PAH [10], this study is the first to implicate heterozygous variation of *ATP13A3* in paediatric-onset cases. The gene encodes a P-type ATPase for which the substrate specificity and biological role remain poorly defined [38], but is a known transmembrane protein that localises to endosomal compartments and is likely involved in polyamine transport, key molecules for cell growth and proliferation [39,40]. Recent functional work demonstrated that *ATP13A3* mRNA is expressed in PASMCs, whilst loss of *ATP13A3* inhibits endothelial cell proliferation and increases apoptosis, consistent with disease initiation models in PAH [10]. The novel c.1148C>A (p.Thr383Lys) variant identified in this study alters a highly conserved amino acid residue located within the actuator domain, which functions to dephosphorylate the catalytic phosphorylation domain (Figure 2). The variant is predicted to disrupt splicing and result in nonsense-mediated decay. Although molecular defects in the *ATP13A3* gene clearly underlie susceptibility to PAH [10,12,20,41], the mechanism of pathogenicity remains to be defined. Further functional analyses are therefore warranted to elucidate whether ATP13A3 loss-of-function represents a molecular mechanism independent to the BMP signalling pathway, and thus a new therapeutic target. 

The WES analysis workflow in this study identified a novel *BMP10* c.247G>A (p.Glu83Lys) missense variant with consistent in silico markers of pathogenicity. The residue is highly conserved across species and is located within the conserved TGF-beta propeptide domain, where a *BMP10* nonsense mutation was previously identified in cPAH [25]. This observation supports accumulating evidence implicating BMP10 loss-of-function in PAH pathogenesis [25,42]. *BMP10* encodes a high affinity ligand that activates the ALK1 and BMPR2 endothelial cell surface receptor within the BMP signalling pathway [43]. The gene product shares 65% amino acid sequence identity with its BMP9 paralogue, encoded by the *GDF2* gene. *GDF2* variant enrichment was recently reported in three large PAH cohorts, strongly implicating the BMP9/BMP10/ALK1/BMPR2 signalling pathway in PAH pathobiology [10,12,20]. Both in vitro and in vivo studies propose a model where BMP9 and BMP10 act interchangeably and with compensatory functionality via ALK1 to regulate endothelial cell migration and proliferation, maintaining endothelial quiescence [44,45]. Based on evidence of sequence homology, mature peptide structural similarities and reported functional redundancy of these gene products, it is proposed that the *BMP10* missense variant reported here may have a similar pathogenic mechanism to those observed in *GDF2*; further exploration on both the genetic and functional level is now warranted.

*PDGFD* missense variants were recently associated with PAH pathogenesis in a case-control analysis of a large, combined cohort [14]. The identification of a novel c.550G>A (p.Glu184Lys) variant of this gene in the cPAH panel analysed here serves to establish *PDGFD* as a contributing factor to childhood disease and provides independent validation of the discovery study. PDGF family signalling pathways play important roles in vascular pathologies, including mediation of remodelling processes that are known to occur in PAH [46]. The plausibility of this gene contributing to PAH risk is further enhanced by cell-type-specific gene expression studies using single cell RNA-seq data, demonstrating high expression in endothelial cells within heart and lung tissue, a key site of disease potentiation in PAH [14]. Of interest, *PDGFD* presents an exciting drug-targeting candidate, with studies of the tyrosine kinase inhibitor imatinib, a known pharmacological inhibitor of PDGF signalling, demonstrating 15-fold reductions in *PDGFD* expression in cardiac tissue [47] and suppression of medial thickening via receptor inhibition in patient-derived in vitro PASMC models [48]. 

The novel c.1069G>A (p.Val357Ile) missense variant identified in *ABCC8* describes an amino acid substitution in the highly conserved transmembrane domain 1 of the mature and functionally important SUR1 product. This finding supports previous investigations that indicate pathogenic variation in *ABCC8* underlies 2–5% of PAH cases, including 10 previous reports of paediatric-onset cases [8,12,24]. The gene encodes the SUR1 subunit of K_ATP_ potassium channels located in cardiac smooth muscle cells. Of note, the variant identified in this study was previously reported to cause hyperinsulinism in compound heterozygosity with a second *ABCC8* allele, although without any clinical signs of PAH [49]. Whole cell electrophysiology and radium efflux assays of reported missense variants have demonstrated loss of K_ATP_ channel activity [24]. Taken together, these data suggest that loss of SUR1-dependent channel function represents a pathogenic mechanism in PAH, although the primary physiological role remains unknown. These findings are of clinical pertinence, as *ABCC8* is considered a possible therapeutic target wherein impaired K_ATP_ activity could be pharmacologically rescued in vitro using diazoxide, a selective potassium-channel opening drug [24]. This may have further implications for the development of diazoxide-induced pulmonary hypertension in children with hyperinsulinaemic hypoglycaemia [50,51].

The *SMAD9* c.1117G>A (p.Val373Ile) variant detected in an HPAH case in this study is predicted to be deleterious by in silico analysis, further supported by familial co-segregation and its location with the C-terminal Mad Homology 2 (MH2) domain, a region of extensive evolutionary conservation. *SMAD1*, *SMAD4* and *SMAD9* encode signalling intermediaries that act as downstream mediators of the BMPR2 pathway. Variants in *SMAD9* were previously identified in three patients [17,52,53], and were recently validated in both adult-onset and paediatric PAH [12,20,22]. Functional analyses of the impact of *SMAD9* variants on SMAD-mediated signalling demonstrated impaired responses to ligand stimulation and reduced transcriptional activation of the downstream BMP target gene, *ID2* [17,52]. Although this variant was deemed a VUS based on our minor allele frequency threshold boundary, it is noteworthy that it was previously detected in an independent patient with PAH. This speaks to the challenges of setting an appropriate MAF cut-off when investigating rare diseases, especially in the context of notable reduced penetrance. Whereas most studies consider an approximation of disease allele frequency equivalent to population disease prevalence, inheritance-based calculations derived from Hardy–Weinberg equilibrium with adjustment for nonpenetrant alleles may be a more appropriate method to avoid false negative findings.

## 5. Conclusions

Taken together, these findings provide data supporting wider locus heterogeneity of paediatric PAH than previously reported and expand the allelic series of variation in known cPAH genetic risk factors. This study supports the inclusion of these genes in screening panels in future work for diagnostic surveillance of paediatric cases. In particular, we provide independent validation of the recently identified risk factors *BMP10* and *PDGFD*, and the first report of an *ATP13A3* variant in a paediatric case, warranting further analysis of understudied cellular pathways key to PAH pathogenesis. Notably, although novel gene detection was beyond the scope of this study in the context of extensive locus heterogeneity, this report emphasises the importance of the rigorous analysis of well-defined case cohorts to delineate the molecular basis of cPAH as a framework for future gene discovery initiatives.

## Figures and Tables

**Table 1:** Variants Identified in Childhood-Onset Pulmonary Arterial Hypertension.

Patient	Sex	Age of Onset	Diagnosis	Variant Identified	Mutation Category	GnomAD MAF	Variant Identifier(s)	CADD Score	SIFT Score	PolyPhen2 (HumVar)	Mutation Taster
P1	M	9 y	APAH–CHD (ASD)	*ATP13A3* (exon 11) NM_024524.4 c.1148C>A (p.Thr383Lys)	Missense *	-	Novel	32	0.001, D	0.991, D	1.0, D
P2	F	3 y	IPAH	*BMP10* (exon 1) NM_014482.3 c.247G>A (p.Glu83Lys)	Missense	0.000009	dbSNP: rs1192957334; Not in ClinVar	28	0.047, D	0.999, D	1.0, D
P3	F	<5 y	IPAH	*PDGFD* (exon 4) NM_025208.5 c.550G>A (p.Glu184Lys)	Missense	-	dbSNP: rs769639743; Not in ClinVar	28.2	0.007, D	0.980, D	0.9998, D
P4	M	19 m	IPAH	*ABCC8* (exon 7) NM_000352.6 c.1069G>A (p.Val357Ile)	Missense	0.000018	dbSNP: rs771392416; Not in ClinVar	23	0.235, T	0.042, B	1.0, D
P5	F	4.5 y	HPAH (mother also affected)	*SMAD9* (exon 6) NM_001127217.3 c.1117G>A (p.Val373Ile)	Missense	0.00013	dbSNP: rs140504903; ClinVar: VCV000311894	27	0.001, D	0.936, D	1.0, D

APAH–CHD: PAH associated with congenital heart disease; ASD: atrial septal defect; B: benign; CADD: Combined Annotation Dependent Depletion; D: damaging (SIFT), probably damaging (PolyPhen2) or disease causing (Mutation Taster); IPAH: idiopathic pulmonary arterial hypertension; HPAH: heritable pulmonary arterial hypertension; MAF: minor allele frequency in gnomAD v2.1.1 (controls); m: months; SIFT: Sorting Independent from Tolerant; T: tolerated; y: years. * The ATP13A3 missense variant in Patient 1 is located within the consensus splice region.
